# EXAMINING THE CHARACTERISTICS OF UNIVERSITY EMPLOYEES AND STUDENTS PROVIDING CARE TO ADULTS WITH ADRD

**DOI:** 10.1093/geroni/igae098.4176

**Published:** 2024-12-31

**Authors:** Jodi L Southerland, Erin Mauck, Shimin Zheng, Matthew Smith

**Affiliations:** East Tennessee State University, Johnson City, Tennessee, United States; East Tennessee State University, Johnson City, Tennessee, United States; East Tennessee State University, Johnson City, Tennessee, United States; Texas A&M University, College Station, Texas, United States

## Abstract

University employee and student caregivers face challenges balancing caregiving with employment and educational responsibilities. With over 4,000 degree-granting institutions in the United States, this represents a growing need for intervention. Data were collected via a Qualtrics survey from university employees and students (n=350) who provided unpaid care to a relative or friend 18 years or older. Participants completed assessments including the Zarit Burden Index, Center for Epidemiologic Studies 4-item Depression Scale, Generalized Anxiety Disorder 2-item scale, and Connor-Davidson Resilience Scale. Chi-square and independent t-tests were employed to compare personal, socio-emotional, and caregiving characteristics between caregivers to people with Alzheimer’s Disease and related dementias (ADRD) and non-ADRD caregivers. Compared to non-ADRD caregivers, the care recipients of ADRD caregivers were older and had more health conditions. ADRD caregivers also had been caregivers for fewer years than non-ADRD caregivers. Although ADRD caregivers were more likely to provide personal care, they also had more caregiving support (e.g., unpaid and paid) than non-ADRD caregivers. Compared to non-ADRD caregivers, ADRD caregivers reported significantly higher caregiver burden (t=-2.39, P=0.02). ADRD caregivers reported a greater need for caregiving resources and were more likely to disclose their caregiving status to university personnel compared to non-ADRD caregivers. Findings highlight the need for university-based resources to support the unique challenges of employee and student ADRD and non-ADRD caregivers. Opportunities exist to introduce programs and services (e.g., respite care, flexible scheduling, problem-solving interventions) to help employees and students succeed in their work/studies, manage caregiver burden, and provide high-quality care to care recipients.

